# Cotton-quality fibers from complexation between anionic and cationic cellulose nanoparticles

**DOI:** 10.1038/s41598-024-69346-y

**Published:** 2024-08-08

**Authors:** Esther E. Jaekel, Guillermo Reyes Torres, Markus Antonietti, Orlando J. Rojas, Svitlana Filonenko

**Affiliations:** 1 Max Planck Institute of Colloids and Interfaces, Am Mühlenberg 1, 14476 Potsdam, Germany; 2 Department of Bioproducts and Biosystems, Aalto University, Vuorimiehentie 1, 00076 Espoo, Finland; 3 VTT Technical Research Centre of Finland, Tampere, Finland; 4 Departments of Chemical and Biological Engineering, The University of British Columbia, 2360 East Mall; Chemistry, 2036 Main Mall, and Wood Science, 2424 Main Mall, Vancouver, BC V6T 1Z3 Canada

**Keywords:** Cellulose fibers, Nanocellulose, Core–shell fibers, Coaxial spinning, Reactive eutectic media, Biomaterials, Nanoscale materials, Structural materials, Green chemistry

## Abstract

Natural polymers are attractive sustainable materials for production of fibers and composite materials. Cotton and flux are traditional plants used to produce textiles with comforting properties while technologies like Viscose, Lyocell and Ioncell-F allowed to extent fiber use into regenerated cellulose from wood. Neither natural nor man-made fibers completely satisfy the needs for cellulose based fabrics boosting development of new approaches to bring more sustainability into the fashion. Technologies like Spinnova are arising based on the spinning of mechanically pretreated cellulose materials with a lower environmental impact though challenged by the fiber quality and strength related to the inconsistency of the mechanical fibers. Nanoscaled cellulose is an excellent solution to improve the consistency of spin fibers, but charges introduced by traditional chemical treatments prevent rebuilding native hydrogen bonding and compromise the mechanical properties especially in wet conditions. We used nanocellulose with low surface charge isolated using reactive eutectic media to spin fibers able to restore the native hydrogen bonding and enable constitutional mechanical strength of cellulose. We performed un-optimized spinning to reveal the intrinsic properties of the fibers and confirmed the preserved strength of wet fibers compliant with the low surface charge enabling further engineering towards cotton-like fabric from wood.

## Introduction

Natural fibers are preferred options for textiles and composite materials due to their performance, renewability, biodegradability, availability, and better positioning to advance net zero efforts. Different forms of cellulose can be sourced from plants, from the highly pure (88–97% cellulose) cotton to those found combined with other molecules such as pectins, proteins, lignin and waxes, including linen (70–75% cellulose) and others. These natural fibers can be spun into filaments used in yarns, ropes fabrics or composite materials^1,2^. The ever increasing demand of cellulose for textiles, however, requires alternatives to cotton, considering the demands of water, land, fertilizers and chemicals used for pesticide, biocide and pathogen control^1^.

Cellulose-based man-made fibers (MMF) are competitive substitutes to cotton, for instance, dissolving-grade wood pulp, a source of viscose (Rayon) and Lyocell, and later Ioncell-F, which are processed by air gap wet spinning following regeneration in a coagulation bath^3–5^. For instance, the Viscose process (Rayon) dissolves cellulose Kraft pulp via derivatization in (highly toxic) carbon disulfide, the Lyocell process uses a more environmentally friendly solvent NMMO (4-methylmorpholine 4-oxide), and Ioncell-F on cellulose dissolution in superbase-based ionic liquid [DBNH][OAc] (1,5-diazabicyclo[4.3.0]non-5-enium acetate)^6–8^. The dissolution and regeneration leads to MMF of given crystal structures (cellulose II polymorphs) in contrast to natural fibers (cellulose I), exhibiting lower primary elastic modulus (80 GPa *vs.* 130 GPa), low stretchability, water absorbency, and exhibiting other structural disadvantages^9,10^. In parallel, there have been many attempts to reprocess wood pulp into cotton-like fabrics, including purely physical Spinnova process that avoids dissolving of the pulp while spinning it with the help of rheology modifiers^11–13^, but the results have not been fully satisfactory^14^.

Cotton fibers have been traditionally considered a preferred choice for the production of woven and nonwoven textiles, given their softness and feel as well as their role is breathable fabrics with good absorbing properties, particularly towards water^15^. Cotton is able to absorb water 24–27 times its own weight retaining the mechanical performance of the fiber^16,17^. The fibers are strong and can stand wear and relatively high temperatures. The excellent physical properties of cotton fibers are the result of the unique multiscale and hierarchical structure of the fibers. As in wood fibers, cellulose in cotton is a primary component of the cell membrane that is formed by a multilayered structure composed of a cuticle, a primary wall (amorphous, less oriented microfibrils), a secondary wall (high crystallinity, ordered layer), and a lumen (hollow structure), conferring cotton its unique properties, including dry strength and stiffness^18^. Cotton fibers though are made of longer crystalline cellulose nanofibers that contain no significant concentrations of lignin (naturally occurring cotton can be seen in brown or green hues caused by lignin or suberin respectively^19,20^). Microscopically cotton fibers look like a hollow ribbon with about 60 twists or convolutions per centimeter^1^. The hollow structure makes cotton fibers light and absorbing, and the twisting and convolutions are responsible for uneven fiber surface that enables yarns of adequate strength to be spun.

To mimic the favorable properties of natural cotton, current approaches have focused on the isolation and use of the primary colloidal building block of plant fibers, namely, high axial aspect cellulose nanofibrils (CNF) or their crystalline domains, cellulose nanocrystals (CNCs). Both of these nanocelluloses can be realigned into cotton-like meso-structures by using different spinning methods enabling fibers with practically reasonable modulus and elasticity. CNC and CNF have both been spun together or separately (wet spinning, dry spinning and flow focusing)^21^ with CNC often combined with a polymer matrix due its low aspect ratio^22^. TEMPO-oxidized CNF (TO-CNF) were first spun into filaments by Iwamoto et al^23^. and the highest reported strengths of such structures have been achieved by Mittal et al. by using a flow-assisted spun CNC into macroscale fibers^24^. Despite the use of nanocellulose featuring a high crystallinity, the mechanical strength of the resultant microfibers is compromised by the charges of the nanoparticles, causing an electrostatic repulsion that prohibits intra crystalline hydrogen bonding.

The present study tackles the detrimental interfacial repulsion by neutralization of moderately charged TO-CNF with CNC bearing positive charges on their reducing ends. CNC is typically isolated using sulfuric acid hydrolysis, which produces high density surface charged nanoparticles (negative sulfate half-esters group) that prevents the close interaction between crystals. Less commonly, hydrochloric acid is used to hydrolyze cellulose to CNC with minimal negative charges and thus prompt to agglomeration that limits processing option^25^. In this study, we used cationic CNC with charged reducing ends isolated using reactive eutectic media as reported by our group^26^. The isolation method allows for CNC to maintain unmodified hydroxyls on the cellulose chain, and introduces charged groups responsible for the colloidal stability of the nanocrystals to reducing ends. Under conditions of low degree of substitution, the oppositely charged CNC and TO-CNF nanoparticles undergo long-range electrostatic interactions, while simultaneously engaging in short-range hydrogen bonding. Alike interactions have been already explored by complexing negatively charged CNC with positively charged biopolymers like carboxymethylcellulose or chitosan, or oppositely charged nanocelluloses^27–29^. However, due to the ionic nature of the interaction in such fibers, their mechanical properties are expected to be particularly sensitive to moisture that would restrict their practical use. Contrary to that, relatively low charges of both cationic CNC and TO-CNF enables stronger hydrogen bonding between nanosized celluloses. In this work, we will point to the role of inter-crystalline hydrogen bonding formation and their influence on the mechanical performance of all-nanocellulose filaments that develop cotton-like properties in both dry and wet conditions.

## Results and discussion

### Coaxial spinning

**Figure 1:**
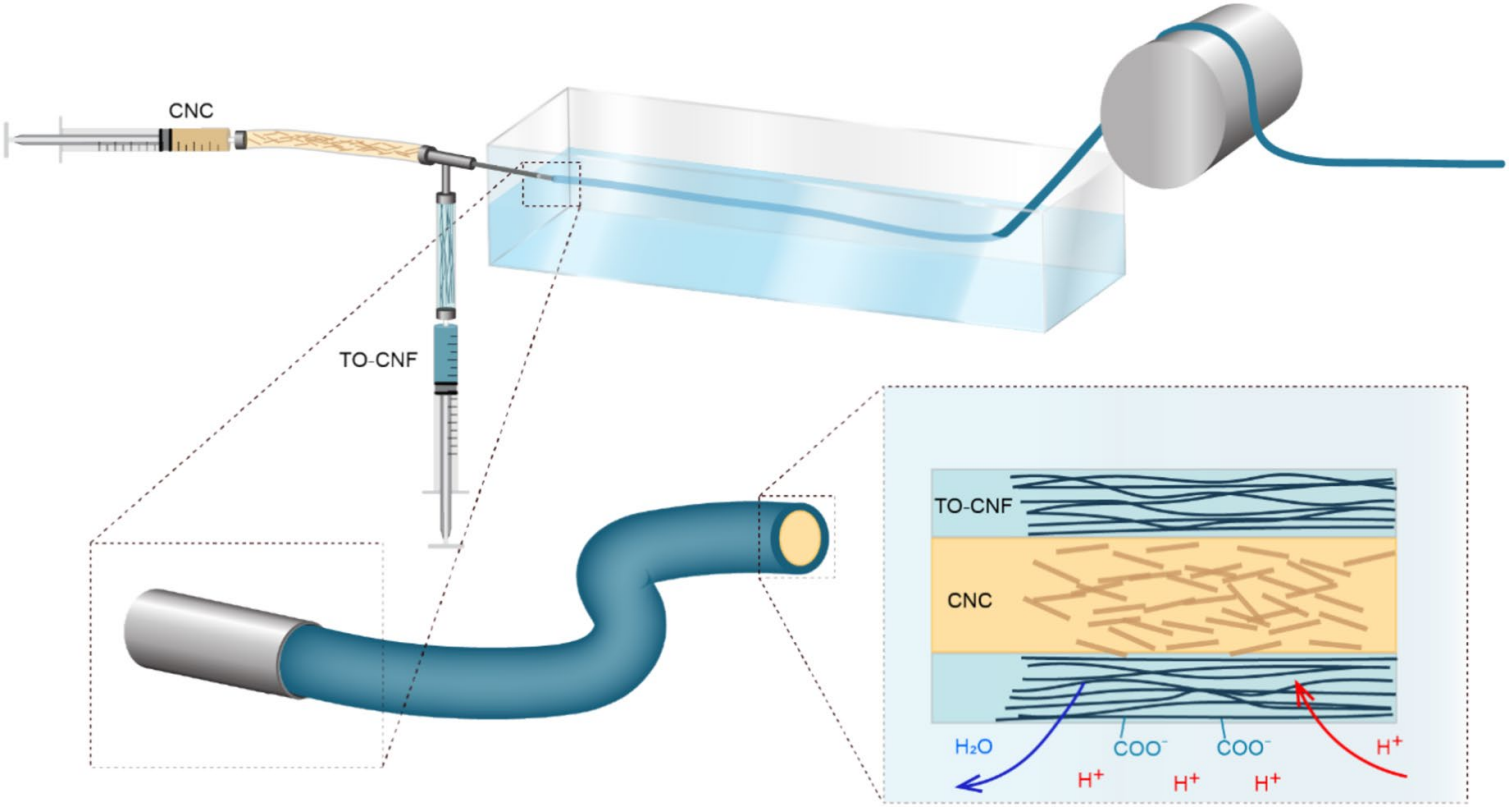
Coaxial spinning setup.

Due to its relatively low aspect ratio, CNC does not develop the entanglement that favors spinning into stable fibers. Coaxial spinning of CNC sheathed into TO-CNF (Fig. 1) was performed as a standard solution to overcome this issue. The filaments were produced after coagulation of the hydrogel filaments formed by TO-CNF and CNC suspended in water, with the latter prepared at given concentrations For spinning, we used cationic cellulose nanocrystals (CNC) isolated using reactive eutectic media and dispersed in DI water to reach solid content of 20 and 30 mg·mL^−1^ (referred CNC_20 and CNC_30, respectively) or in 5vol% acetic acid solution with the CNC content of 15 mg·mL^-1^ (referred CNC_acid). Increasing concentration of CNC in dispersion was aimed to test the optimal inside filling of the TO-CNF outer fiber, while acidic pH provide the maximal ionization of the cationic CNC. The core (CNC)-shell (TO-CNF) filaments were produce by the effect of complexation of the two phases, which developed a strong interface^30^.

**Figure 2:**
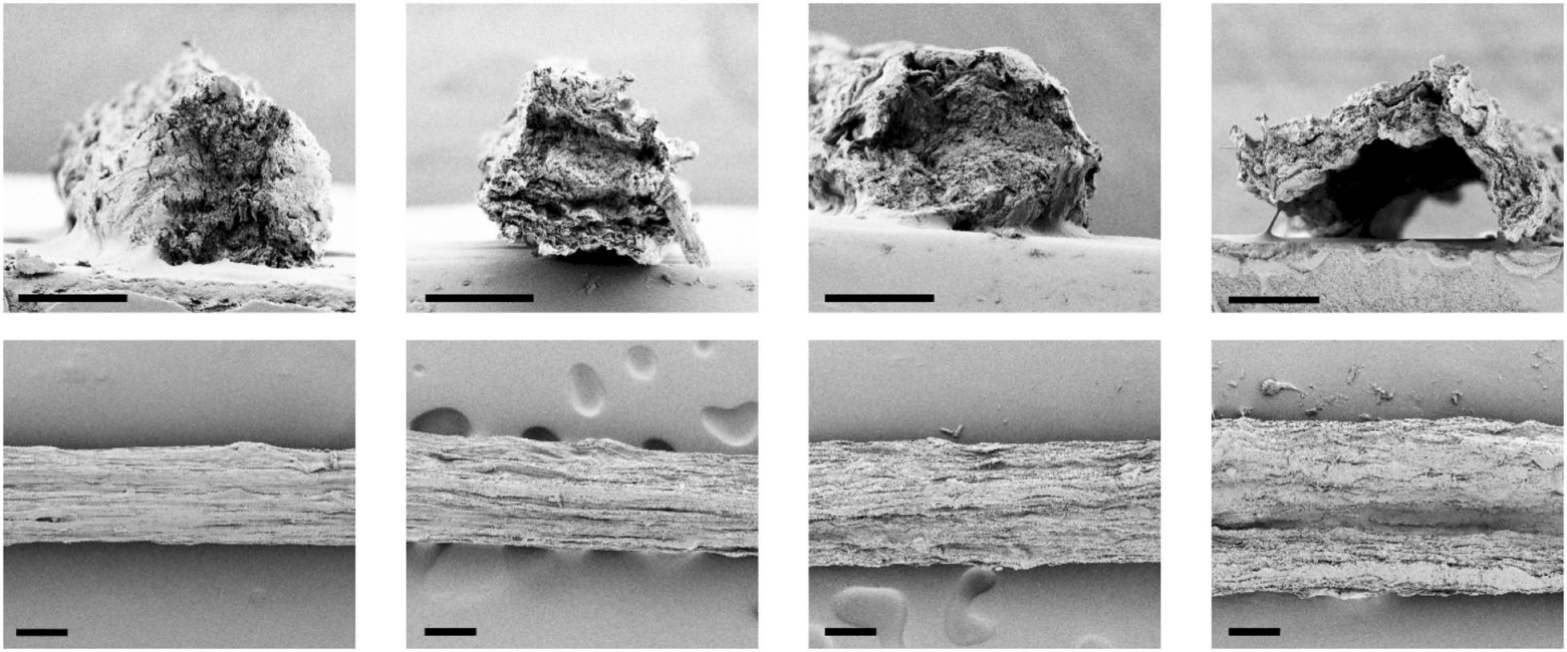
SEM of cross sections and top-views of fibers prepared via coaxial spinning of cationic CNC sheathed with TO-CNF: (**a**) reference TO-CNF used as core and shell components for the filament; spinning of CNC_20 (**b**), CNC_30 (**c**) and CNC_acid (**d**) as a core. Scale bar: 100 µm.

The SEM images of the filament cross sections revealed striking differences between the spun fibers produced with an acidic CNC core and all the others (Fig. 2). CNC_acid formed a flat, sheet-like filament, while the reference as well as the other CNC-containing fibers have an approximately circular cross-section. The lower CNC concentration in the acidic sample alone (15 mg·mL^−1^ *vs.* 20 and 30 mg·mL^−1^) cannot explain this difference. The acidic medium in the spinning CNC dispersion appears to drive attraction of the cationic cellulose components to the outer interface, also pointing to improved mass transport of the ionized nanocrystals. This in a good accordance with the highest degree of ionization of CNC at pH ca. 3 (Figure S1). Stronger attraction between the ionized cationic CNC and negatively charged TO-CNF primarily resulted in a hollow fiber that was flattened upon collecting on the winder. Interestingly, this morphology is very similar to the cotton fibers, which are biosynthesized as a hollow fiber that flattens with seed drying and maturization. Consequently, filaments with the CNC_acid core were the only ones capable of temporarily support a 1-kg load (SI video).

### Colloidal properties of TOCNF/CNC mixtures

**Figure 3:**
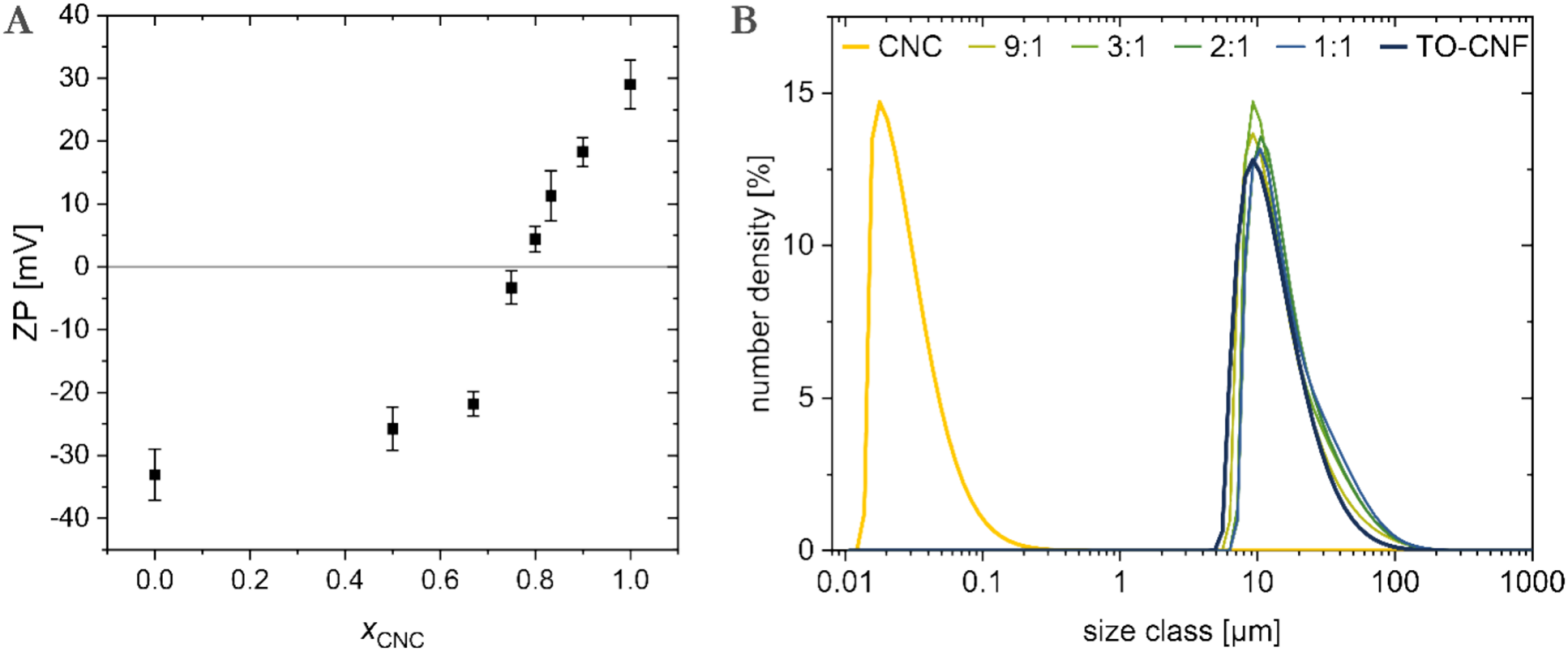
(**A**) Zeta Potential (ZP) of mixtures of TO-CNF with CNC plotted against the ratio of CNC ($${x}_{CNC})$$ in the mixture, (**B**) size distribution determined via SLS of CNC and TO-CNF, ratio given as CNC:TO-CNF.

Cationic CNC and anionic TO-CNF are expected to interact upon mixing due to electrostatic attraction between the opposite charges. The negative charges of TO-CNF are related to the carboxylic groups introduced by oxidation of hydroxyl groups predominately at the C6 position of glucose units. The positive charge is introduced to CNC by reductive amination of carbonyl group on the reductive end of cellulose polymer chain (Eq. 1). To investigate the interaction between differently charged nanocelluloses, the zeta potential of the CNC and TO-CNF in aqueos suspensions at different ratios is measured (total concentration of 1 mg·mL^-1^). The zeta potential of the single-component suspensions, TO-CNF and CNC were -33 mV and + 29 mV, respectively (Fig. 3). The mixed nanocelluloses at given mass ratio of CNC, ${\text{x}}_{\text{CNC}}$, showed zeta potential between the individual values, with a shallow initial slope of the ZP versus ${\text{x}}_{\text{CNC}}$ followed by a steeper increase at ${\text{x}}_{\text{CNC}}>0.7$ until reaching a point of zero net charge around ${\text{x}}_{\text{CNC}}=0.8$ and reaching the charge of the pure CNC above the ratio. Size distribution curves of the pure nanocelluloses and their mixtures (Fig. 3 B) demonstrates that CNC completely adsorb on the CNF at every tested ratio, as the absence of a signal in the nanometer range suggests. This result demonstrates the complexation by electrostatic interactions between the two nanocellulose components.

Equation 1 Positive charge of the cationic CNC is due to quaternary ammonia groups at the reducing end of cellulose chain; TEMPO oxidation introduces carboxylic groups across the cellulose chain on C6 position of glucose units. Consequently, the cationic cellulose has the highest charge at lower pH values, while carboxylic groups are ionized at higher pH. 1

### Mechanical performance of the coaxial filaments

**Figure 4:**
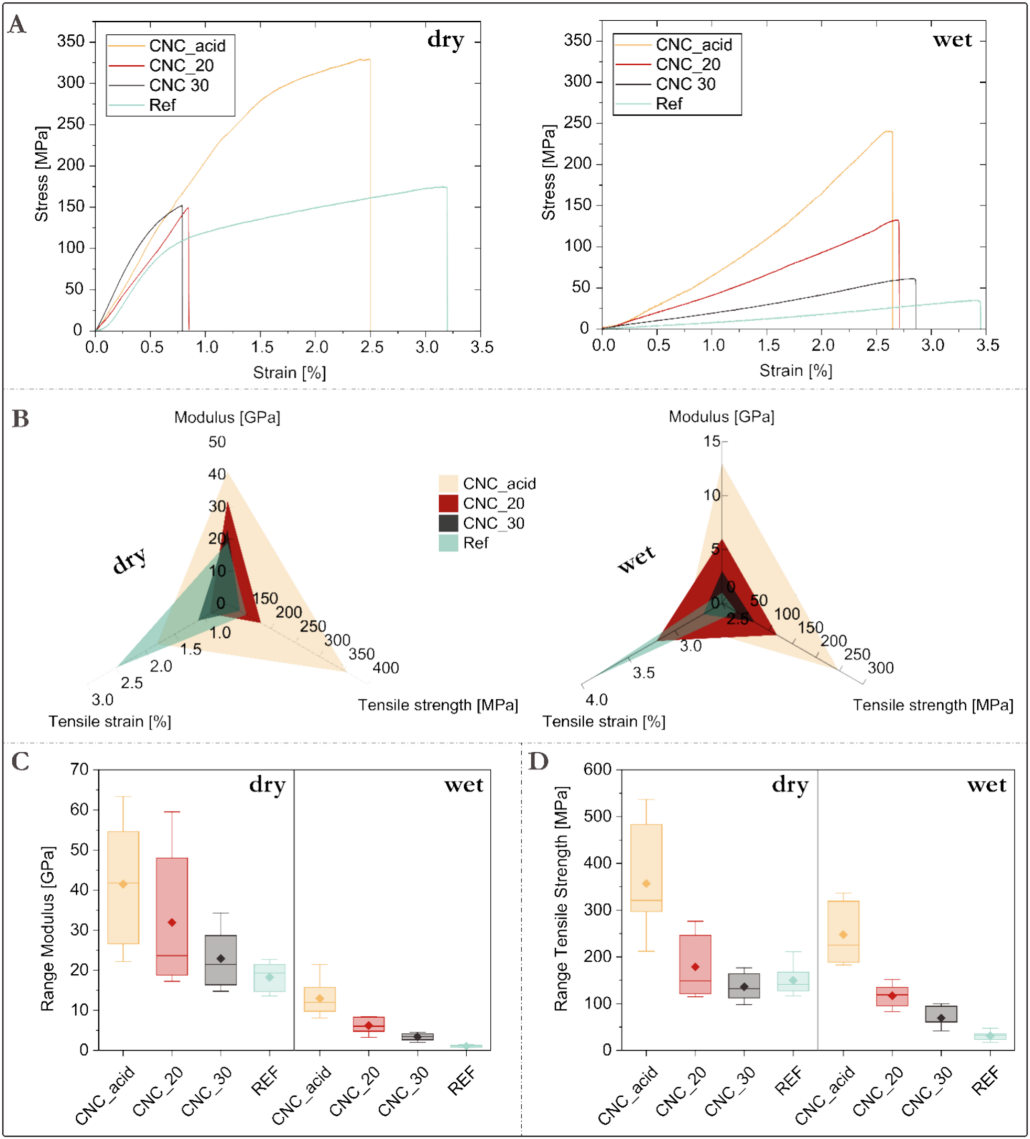
Mechanical properties of the tested yarns averaged over 8 samples.

Tensile strength measurements were performed in dry and wet conditions (Fig. 4). Expectedly, the CNC core increased the stiffness of the filaments due to its rigid rod-like nature, amounting to an increase from 28 to 64% compared to the reference (single-component TO-CNF filaments). The filaments carrying the CNC_acid core exhibited significantly increased modulus (41 GPa) and tensile strength (357 MPa) compared to the reference spun only from TO-CNF filaments (18 GPa and 149 MPa respectively). The difference is even more striking in wet conditions. The reference TO-CNF filaments decreases from 19 to 1 GPa in modulus, equivalent to a 94% decrease, and the modulus of the coaxial filament formed with CNC_acid core decreased by only 68% to 13 GPa exhibiting an extraordinary for a cellulose filament wet strength. The wet tensile strength of CNC_acid decreased by 30% to 148 MPa. The reference, on the other hand, exhibits a decrease of 79% dropping to 31 MPa. Higher concentrations of CNC in water applied as a core resulted in a limited increase on tensile strength and modulus though outperforming the single component TO-CNF reference filaments. Compared to other synthetic fibers, the produced filaments reach higher specific modulus and strength for instance compared to regenerated alkali cellulose with a modulus of about 12 GPa and tensile strengths up to 110 MPa^31^ and one order of magnitude superior regarding Young modulus and tensile strength compared to man-made fibers such as Nylon, Rayon, polyethylene (PE), and cellulose acetate^32–35^ and similar mechanical performance when comparing to state-of-the-art Ioncell® fibers^36^.

Statistical analysis shows that the increase in modulus and strength in CNC_acid was significant compared to the other samples. The differences among the values is generally higher in the CNC-containing fibers than in the reference (single component TO-CNF filament). This could be related to the un-optimized flow conditions for CNC leading to the local structural differences in the filaments. Better alignment of TO-CNF is supported by the higher degree of orientation (Table 1).

**Table 1:** Orientation parameters and density of the fibers.

Sample	Degree of orientation (%)	Angle (°)	Herman factor	Density [g/mL]
*REF*	35 (1)	1**77** (0.8)	0.501 (0.007)	1.50 ± 0.33
*CNC_acid*	27 (0)	176.7 (0.6)	0.448 (0.001)	1.66 ± 0.23
*CNC_20*	32 (1)	178.1 (0.9)	0.487 (0.009)	1.04 ± 0.32
*CNC_30*	30 (1)	172.1 (0.9)	0.464 (0.008)	1.20 ± 0.23

### Orientation in the filaments and their crystal structure

**Figure 5:**
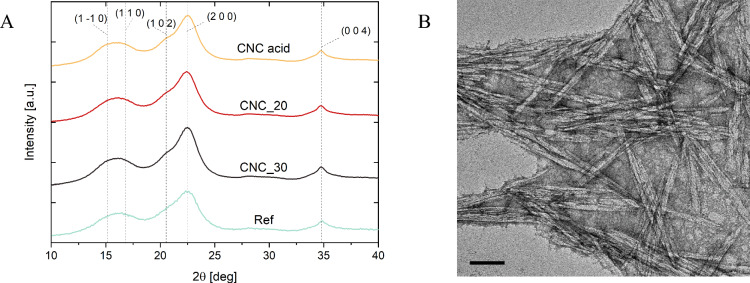
WAXS diffractograms of spun fibers (**A**); TEM micrographs of cationic CNC (**B**).

Using Wide Angle X-ray Scattering, the crystallite orientation in the filament was investigated (Fig. 5A). The comparison of the orientation properties shows that crystallites in the filaments showed a Herman orientation factor in the range of 0.4–0.5, below those for the natural cotton (ca. 0.7) or engineered nanocellulose fibers^14,37^. This is expected from the spinning set-up of the experiment where drawing of the fibers was intentionally avoided to reveal the influence of the inherent interactions between the cationic CNC and anionic TO-CNF on the strength of produced filaments. In fact, CNC_acid were mostly disordered (no flow assisted or shear-induced orientation). Figure 5B demonstrates random orientation of CNC upon drying on TEM grid. The low surface charge of the CNC makes disordered orientation of the nanocrystals preferable also in the filament. However, the strength developed by this latter filament indicate the role of electrostatic interaction, leading to a higher strength that cannot be attributed to a higher orientation of CNC inside of the filament, i.e. in the direction of strain. As in the whole filament about a third of the main axis modulus of the CNC was reached, we can state to be close to theoretical expectations: that is the fiber is able to tap on the primary modulus of the building blocks, without excessive interface dissipation. This supports beneficial fortification of the TO-CNF with crystalline CNC.

To investigate other reasons for the higher strength of the CNC_acid fiber, the fiber densities were measured. While CNC_20 and CNC_30 have significantly lower density than the TO-CNF (i.e. the cellulose constructs are porous), the density of CNC_acid is indeed around 10% higher than the CNF reference higher than bulk cellulose and of the order of the density of the CNC^38^. The dense packing can point to the closer interaction between the nanocrystals and nanofibers.

Side view on SEM (Fig. 2), all fibers indicate a similar degree of overall orientation of the substructure along the fiber, giving a visual idea on what a Hermann factor of 0.5 means.

## Conclusions

We explored the possibility to generate a synthetic cotton-like cellulose fiber by electrostatic self-assembly of positively charges cellulose nanocrystals and negatively charged cellulose fibers. In the current setup, rather weakly organized fibers could be obtained, which however realized about 30% of the elemental Young modulus on the fiber level. Optimal fiber spinning composition with some acetic acid in the spinning medium created dense fibers, which by morphology were interpreted to be collapse hollow fibers. Unexpectedly, the fibers showed a striking wet strength, indicating the high stability of electrostatic and hydrogen binding also in water, outperforming all classical reference measurements. The reported method expands the possibilities of manipulating nanocellulose fibers into high-strength materials and allows dreaming of replacing cotton by ordinary cellulose pulp.

## Experimental

### Materials

Ammonim formate, lactic acid, pre-fibrillated never-dried Kraft pulp (MERCER), acetic acid, ethanol.

### Fabrication of cationic CNCs

Cationic CNCs were obtained using our previously reported one-pot extraction method that employs a reactive eutectic medium (REM) composed of ammonium formate and lactic acid in a molar ratio of 2:1. Briefly, a pre-fibrillated never-dried Kraft pulp provided by MERCER was mixed with the REM to obtain 10 wt% of solid content and reacted in a stirred autoclave at 160 °C for 2 h. The solid cellulosic product was separated from the REM and washed via centrifugation and re-dispersion of the precipitate using a sequence of acetic acid (5 vol%) and ethanol. After the supernatant remained colorless, the solvent was exchanged to either water (CNC_20 and CNC_30) or 50 mM acetic acid (CNC_acid). The dispersions were homogenized using a pressure cell homogenizer and the concentration of the CNCs was adjusted to 30 mg/mL (CNC_30), 20 mg/mL (CNC_20) and 15 mg/mL (CNC_acid) with DI water or acetic acid solution respectively.

### Fabrication of TEMPO-oxidized CNFs

TO-CNF were fabricated from Kraft bleached birch pulp from a Finnish pulp mill UPM (kappa number 1; DP 4700; fines-free) according to the method of Isogai et al.^39^ following the protocol by Reyes et al.^30^.

### Co-axial spinning

The TO-CNF hydrogel (2 wt%) and the CNC dispersion (1.5 wt%, 2 wt% or 3 wt%) were homogenized and de-aired in a planetary centrifugal mixer (THINKY AR-250, JAPAN) individually before being transferred into the pumping syringes (Henke Sass Wolf, 60 mL, Luer Lock, soft jet; pumps: CHEMYX, model NEXUS 6000, and CHEMYX, model FUSION 6000, USA), which were connected to a coaxial dispensing needle (Ramé-Hart Instrument CO, shell needle gauge 15 outer diameter Φo = 1.83 mm, and inner diameter Φi = 1.37 mm, and core needle outer diameter Φo = 0.889 mm, and inner diameter Φi = 0.584 mm). The pumps were operated at flows of 2 mL $\cdot$ min^−1^ (shell) and 0.6 mL $\cdot$ min^−1^ (core). The filament was spun into a stainless-steel coagulation bath filled with 0.01 M HCl leaving an air gap of 2 cm between needle and bath. The acidic conditions cause coagulation of the TO-CNF based on the protonation of the carboxyl groups. The spun filament was taken up on a stainless steel winder (6 cm diameter) rotating at 67.5 cm/min, resulting in a draw ratio ${\text{D}}_{\text{w}}$ of 1.15. The setup is illustrated Fig. 1.

### Mechanical and density testing

Tensile Test and densities were studied using an Automatic single fiber testing device FAVIGRAPH (Textechno Herbert Stein GmbH & Co. KG, Mönchengladbach, Germany). Samples were prepared and analyzed according to the ASTM D3822/D3822M standard. Samples were stored and tested in a conditioned room at 50% R.H at 23 °C using six replicas per sample. Filament diameters were calculated SEM equivalent cross section areas. The apparent density was calculated, assuming that each filament has cylindrical morphology. The apparent porosity was calculated with reference to the reported density consequently (1.55 g · cm^−3^) of pure or crystalline cellulose I^40^.

### Statistic analysis

Spun fibers moduli, tensile strength and strain were studied with one-way ANOVA analysis and Tukey test *p* < 0.05. The statistical analysis results, including box charts (Fig. 3), and Overall ANOVA analysis with the Tukey test are presented in supporting information. Errors bars are presented as standard deviations.

### WAXS measurements

X-ray crystallography data was obtained using a small-angle and wide-angle X-ray scattering (SAXS/WASX) device (Xenocs, Xeuss 3.0, U.K.) bench beamline equipment. The generator worked at 45 kV and 200 mA with Cu Kα radiation (λ = 0.15418 nm). The diffractograms were recorded in 2*θ* range from 10° to 40° with the step size of 0.02°. The filaments were measured in a position perpendicular to the X ray beam using the Xenocs solid sample holder (as shown in the figure). The background signal correction was made by subtracting the sample diffractogram data with the corresponding reference signal (without sample). The degree of orientation was calculated using *Herman’s factor.*^37^ Every sample was measured in three different positions, and the deconvolution and parameter calculation was performed following a reported procedure^41^. The degree of orientation and the Herman orientation factor were determined (Table 1)^37^. A degree of orientation of 1 means perfect alignment of the fibers (crystallites) to the long axis of the fiber, while 0 means isotropic fiber distribution.

### TGA

Thermogravimetric analysis (TGA) was performed using a Thermo microbalance (TG 209 F1 Libra, Netzsch, Selb, Germany) under a constant nitrogen flow of 100 mL $\cdot$ min^−1^. 10 mg of the dry filaments were filled into an aluminum crucible and heated from 25 to 600 °C with a heating rate of 10 °C $\cdot$ min^−1^.

### SEM

Scanning electron microscopy (SEM) images were acquired on a Gemini 1550 (Zeiss AG, Oberkochen, Germany) using an accelerating voltage of 3.00 kV. Fiber cross sections were prepared by freezing the fiber in liquid nitrogen before rapidly breaking it.

### Supplementary Information

Supplementary Information.

## Data Availability

Data is provided within the manuscript or supplementary information files.
