# Supplementary material for: Cotton-quality fibers from complexation between anionic and cationic cellulose nanoparticles
Source: Sci Rep. 2024 Aug 8;14:18406. doi: 10.1038/s41598-024-69346-y (PMC11310312; doi:10.1038/s41598-024-69346-y)
Supplement: Supplementary file 1 — Supplementary Information. [file 41598_2024_69346_MOESM1_ESM.docx]

**Supplementary materials**


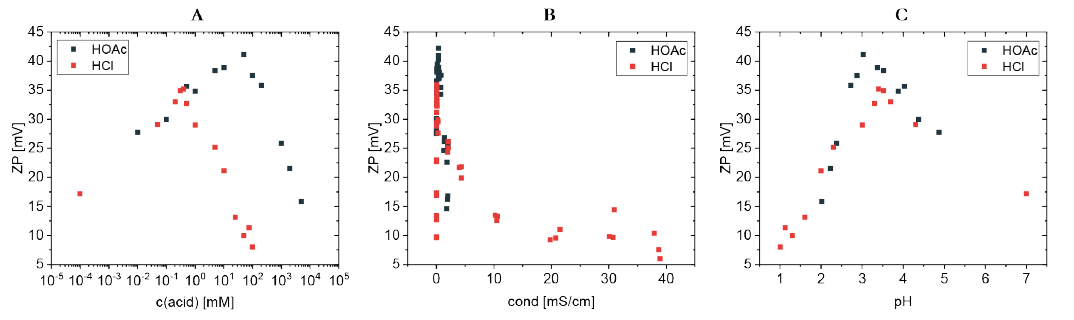


Figure S1. Titration curve for cationic CNC with acetic (HOAc) or hydrochloric acid (HCl). Equivalence point at pH ca. 3 for HOAc and ca. 3,5 for HCl.

Thermal properties


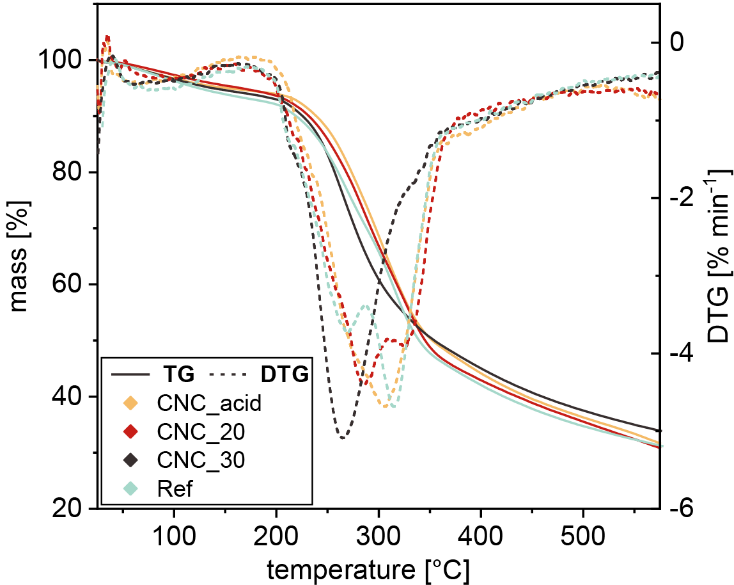
TGA reveals no significant differences in the thermal stability of most of the investigated fibers. The mass loss peak of CNC_30 appears shifted towards lower temperature. The lower degradation point could be related to a lower density and higher specific surface area, promoting the evaporation of volatile fragments (compare *Table 1*).

Figure S 2. TGA and DTG curves of the fabricated fibers


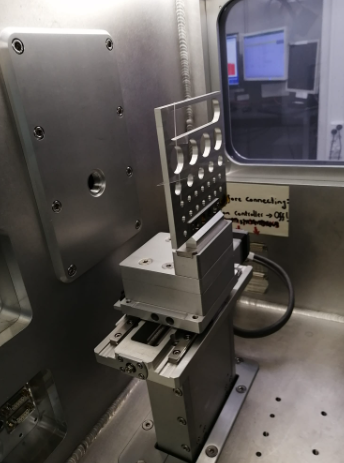


Figure S 3. Setup for the WAXS measurements
